# SteadEye-Head—Improving MARG-Sensor Based Head Orientation Measurements Through Eye Tracking Data

**DOI:** 10.3390/s20102759

**Published:** 2020-05-12

**Authors:** Lukas Wöhle, Marion Gebhard

**Affiliations:** Group of Sensors and Actuators, Department of Electrical Engineering and Applied Sciences, Westphalian University of Applied Sciences, 45877 Gelsenkirchen, Germany; marion.gebhard@w-hs.de

**Keywords:** data fusion, MARG, IMU, eye tracker, self-contained, head motion measurement

## Abstract

This paper presents the use of eye tracking data in Magnetic AngularRate Gravity (MARG)-sensor based head orientation estimation. The approach presented here can be deployed in any motion measurement that includes MARG and eye tracking sensors (e.g., rehabilitation robotics or medical diagnostics). The challenge in these mostly indoor applications is the presence of magnetic field disturbances at the location of the MARG-sensor. In this work, eye tracking data (visual fixations) are used to enable zero orientation change updates in the MARG-sensor data fusion chain. The approach is based on a MARG-sensor data fusion filter, an online visual fixation detection algorithm as well as a dynamic angular rate threshold estimation for low latency and adaptive head motion noise parameterization. In this work we use an adaptation of Madgwicks gradient descent filter for MARG-sensor data fusion, but the approach could be used with any other data fusion process. The presented approach does not rely on additional stationary or local environmental references and is therefore self-contained. The proposed system is benchmarked against a Qualisys motion capture system, a gold standard in human motion analysis, showing improved heading accuracy for the MARG-sensor data fusion up to a factor of 0.5 while magnetic disturbance is present.

## 1. Introduction

Measuring head motion for medical diagnostics, virtual or augmented reality applications or human-machine collaboration usually involves multimodal sensorized interfaces to estimate motion and generate an appropriate control input for the desired application. In the context of human-machine collaboration and direct interaction with assistive technologies these interfaces are often designed to be used hands-free [1,2,3]. Such an interface needs to be precise, robust and fail safe. The research and development of new interaction technologies is in demand. A promising hands-free sensing modality involves Magnetic AngularRate Gravity (MARG)-sensors to estimate orientation of the operators head to interact with or teleoperate a robotic system [1]. In general MARG-sensors consist of a tri-axis accelerometer, tri-axis gyroscope as well as a tri-axis magnetometer. Such a sensor can also be termed inertial measurement unit (IMU) if it does not feature the tri-axis magnetometer. The orientation estimation from these sensors is based on the integration of the gyroscope raw angular rate data. This raw signal suffers from various noise terms that need to be taken care of, especially the dc-bias [4]. Even if the dc-bias of the gyroscope is set to zero at the MEMS-fab, there is a dc-bias that depends on sensor type, packaging and temperature, which in turn leads to a drift of the integrated gyroscope data [5]. This drift is usually corrected by the underlying data fusion process. Most common algorithms use the absolute references, direction of gravity and geomagnetic field to reduce orientation drift introduced through the gyroscope measurements [4,6]. The influence of permanent magnets, that is, hard iron effect, and magnetizable materials, that is, soft iron effect, in the direct neighborhood of the MARG-sensor will cause superpositions of the surrounding magnetic field. The distorted magnetic field can no longer be used to correct for dc-bias errors in the heading estimate (yaw-axis). During magnetic disturbances the MARG-sensor relies on gyroscope data only and will over time result in a drift in the heading estimate because of the accumulated errors if not corrected somehow [7,8].

This paper presents a novel approach to reduce heading estimation errors of head movement measurements by functional combination of mobile eye tracking and a head worn MARG-sensor. The approach utilizes the physiological connection between eye and head movements to identify static and dynamic motion phases. The eye tracking glasses are used to track visual fixations which indicate static phases. This indication is used for zero orientation change updates in a MARG-sensor data fusion algorithm. The approach relies on an infrared based eye camera only and does not need a scene or world camera and therefore no eye to world camera calibration. The presented approach is decoupled from most surrounding environmental conditions.

Human-robot collaboration in industrial production as well as rehabilitation robotics are applications that benefit from the proposed approach. These applications are mostly indoor and introduce potential magnetic interference at the location of the MARG-sensor. The work presented here not only enhances robustness of heading estimate of the head orientation measurements, it also presents a self-contained device mostly decoupled from varying visual markers and lightning conditions in the surrounding scenery.

### 1.1. State of the Art in MARG Based Orientation Measurements

Many algorithms exist to fulfill orientation estimation for head motion tracking. The Kalman filter has become the de facto standard for industrial and research applications [9,10]. However, various fast and lightweight data fusion methods for orientation estimation have been developed to reduce computational load while keeping orientation errors at a reasonable level. A famous method was introduced by Madgwick et al. in 2011 [11]. This method is based on the gradient descent algorithm and has gained popularity due to its fast and effective implementation on a microcontroller. Unfortunately MARG-sensors are exposed to magnetic field disturbance, termed soft- or hard-iron effects, resulting in incorrect orientation estimation [7]. This error scales with respect to the distance between the sensor and source of magnetic disturbance and magnetic properties of the source, for example, 35–50${}^{\circ }$ error near large ferromagnetic metal objects or the floor (indoor) [12]. Recent approaches try to overcome these magnetic disturbances by software, for example, online gyroscope bias estimation [13] or fast online magnetometer calibration [14]. These software based corrections do not require additional hardware nor other sources of reference but might require certain motion conditions. For example online magnetometer calibration approaches are usually based on a sampling of sparse 3D magnetometer data points to adapt the calibration matrix, which might not be possible in every situation due to fast changing magnetic field values or the rather small motion space of the head. The use of other hardware, for example, visual odometry, usually provides a decent source of reference for the heading estimate but depends on the surrounding environmental conditions, for example, structured environment, reasonable lighting conditions and small relative motion in the scenery [15]. These conditions can not always be guaranteed especially in the context of human-machine collaboration which will feature a lot of relative motion from the robotic system and heavy dynamic magnetic disturbances due to the robots metal-links and motor-joints.

### 1.2. State of the Art in Eye Tracking

The analysis of the direction or the location of interest of a user through eye tracking is key for many applications in various fields, that is, human-computer interfaces (gaze mouse), human-machine interfaces, medical diagnostic and many more [16]. Therefore, fast and reliable eye tracking devices and software have been heavily researched [17]. Eye trackers can be separated into stationary or mobile devices. Stationary eye trackers are fixed in position referenced to the world frame. The devices’ camera observes the users eyes and maps the tracked gaze to a defined surface (e.g., screen monitor) [18]. Mobile devices on the other hand usually consist of a frame, that is worn like a pair of glasses, a mono- or binocular eye camera fixed to the frame monitoring the pupil and a world camera to merge calibrated pupil positions to a gaze point in the world frame [17]. Furthermore, modern mobile eye tracking devices either feature a MARG-sensor or an IMU-sensor or the eye tracker can be extended by a custom or third-party sensor board. In this work, we use a monocular mobile eye tracker that gained popularity in the research community over the past years due to the open source software and affordable pricing [19].

## 2. Working Principle

**Figure 1:**
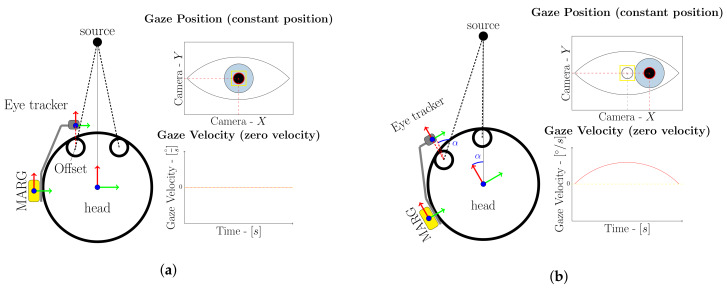
Coordinate systems between eye tracker, Magnetic AngularRate Gravity (MARG)-sensor and head when fixating an object (**a**) without and (**b**) with head motion. The condition of fixation of the object results in a stable gaze position. Possible motion of the pupil during fixations itself, so called microsaccades, is very small (0.2° at a duration of 20–30 ms) and can be neglected (yellow boundary box).

The proposed work is based on the physiological relationship between eye and head rotations during visual fixations of stationary objects. The eyes of a human are centered in a fixed axis of rotation inside the head and are therefore naturally affected by head rotations. Visual fixations of objects will result in small or nonsignificant rotation of the eyeball during stationary motion phases, see Figure 1a. A rotation of the head during visual fixation of a stationary object however, will result in an opposite rotation of the eyeball due to the vestibo-ocular reflex, stabilizing the visual scenery [20], see Figure 1b. The physiological relationship between head and eye rotations therefore represents a natural indicator for head rotation and can be used to support head orientation measurements.

A mobile eye tracker is used to measure the above mentioned relationship and utilize this indicator for MARG-sensor based head orientation measurements. The method assumes that the MARG-sensor is fixed in position with respect to the eye tracker frame, for example, attached to it. The mobile eye tracker is worn by the user and should be adjusted in a way that prevents heavy slippage during head motion, for example, through an eye wear strap which is common practice in mobile eye tracking. From the setup given in Figure 1, the following constraints can be derived.

The coordinate system of the mobile eye tracker and MARG-sensor share a common reference frame with the users head and rotate conjointly, see Figure 1. Every rotation of the head is directly coupled with the rotation of the eye-tracker frame and MARG- or IMU-sensor. This rotation will result in a change of the pupil position in the eye camera image. This is either due to a voluntary change in the visual fixation or a head movement. In contrast to head rotation based changes of the pupil position, a change of visual fixation is usually coupled with high angular velocity of the eyeball due to a significant pupil position change of consecutive eye camera images. If the visual target is moving while the head is stationary or the fixation changes to another visual target, there will be a significant change in pupil position, due to the given coordinate system setup. The eye tracker camera is in a fixed position with respect to the head coordinate system. If the eyes follow a moving target or switch the visual fixation the pupil position changes within consecutive frames because the target changes its position with respect to the head and eye tracker coordinate system. Changes in the pupil position will therefore always indicate motion, whether it is introduced through head motion or voluntary eye motion. Near zero changes in the pupil position between eye tracker camera images however indicate near zero head rotation with one possible exception from this assumption. If a visual fixation stays on a moving target while the head is rotating at the same rotational speed at which the target is moving, all local coordinate systems do keep their relative positions between each other. This would result in a no motion classification from the pupil position change criteria. In this kind of situation the pupil position does not change with respect to the eye tracker coordinate system since the head and target coordinate system do not change their relative positions and orientations between each other. A MARG- or IMU-sensor however does measure motion related to the world coordinate system and will therefore measure a change in the orientation between the world and eye tracker coordinate system. The rotational velocity of the motion, or in other words the change of orientation between coordinate systems, needs to exceed a minimum threshold to distinguish the motion from gyroscope noise during these special phases.

The approach presented here uses this pupil motion description under visual fixation of an object to reduce the drift effect at stationary phases and therefore improve the MARG-sensor based heading estimate. Since all local coordinate systems are in a fixed position towards each other, every simultaneous measured movement or motion is caused by head rotations. Every significat pupil position change indicates motion, either from head rotation or eye rotatation. Zero or no rotation however, is indicated by every visual fixation, independent of the total fixation time, that results in near zero change in the pupil position and angular velocity.

## 3. Data Fusion Process

In this work it is proposed to support MARG-sensor based heading estimates by zero rotation updates measured and indicated by visual fixation detection. The detection of visual fixation (given in subsection A) is used to feed the previous estimate of heading from the MARG-sensor fusion process (given in subsection B) recursively to the filter itself to reduce accumulation of gyroscope bias related heading errors. We calculate an IMU heading vector ${}^{N}{\vec{N}}_{IMU,k}$ based on the previous estimate of the heading that represents the direction of a horizontalized heading vector in the North East Down (NED) frame. This heading vector can be used as a complete substitute to the magnetometer based horizontalized north direction vector in the MARG-equation of an adapated form of Madgwick’s Gradient Descent filter stage.

Synchronization of both systems is achieved through timestamp based message filtering. The data of the mobile eye tracker as well as the used MARG- or IMU-sensor should be accessible in real time by the manufacturers application programming interface (API) and provide a timestamp that can be used for synchronization processes, for example, using the message filter package from the robot operating system (ROS) framework. An angular rate threshold based switching can be either implemented on the MARG-sensor or host computer to account for possible latency issues between both systems. This threshold is based on the median head motion noise in static motion phases indicated by visual fixations. If the gyroscope raw signal exceeds the median noise level, the zero orientation update is turned off. This median noise threshold is also used to address the special case that the pupil position does not change while the head and eyes are following a moving target at the same rotational speed. During these special motions the magnitude of the measured angular rate will exceed the median gyroscope threshold which in turn disables the zero orientation update.

### 3.1. Visual Zero Rotation Detection

The trigger signal for the zero rotation update is based on an online visual fixation detection algorithm that utilizes dispersion (spatial movement, $th_{d}$) and duration ($th_{t}$) thresholds to identify fixations. These thresholds define the total amount of allowed gaze or pupil position differences (Δp) between time successive eye camera images (Δt). The algorithm utilizes a sliding window which length is determined by the duration threshold $th_{t}$ and sampling frequency. Dispersion *p* is calculated as the sum of the differences between consecutive pupil positions

(1) Δp=[(max(x)−min(x)+(max(y)−min(y)],

where *x* and *y* are the eye tracker cameras pixel positions. The dispersion is compared to the maximum dispersion threshold $th_{d}$. Fixations are identified as such if the dispersion stays below $th_{d}$. This results in an expansion of the window to the right until the dispersion exceeds this threshold. If no fixation is detected the window does not expand but moves forward in time [21].

This kind of algorithm has proven to be very accurate and robust regarding online fixation identification but needs careful parameter setting [21]. While the visual fixation stays on a target and inside the dispersion threshold boundaries, the head is assumed to be stationary. The threshold parameter ratings for the magnitude of dispersion in time is given due to involuntary movement, for example, microsaccades and tremor. However, these involuntary movements usually consist of rather small duration in the range of 20–30 ms and amplitudes peaking in a visual angle of 0.2° [22]. The fixation detection parameters should be chosen in a way that fixations are still detected even in the presence of microsaccades and tremor. A fixation is identified and labeled as such, as soon as the fixation duration threshold is reached. Upon this a trigger signal ($S_{t}$) is emitted indicating a zero orientation update cycle for the MARG-sensor data fusion process.

(2) $$S_{t}=\left\{\begin{array}{c} 1,\;\Delta p\leq th_{d}\wedge \Delta t\leq th_{t}\\ 0 \end{array}\right.$$

The trigger starts an acquisition cycle that stores gyroscope raw data while the fixation holds true. When a sufficient amount of gyroscope samples has been recorded, updated motion noise parameters are sent to the MARG-sensor to update the threshold to account for desynchronization and latency issues between both systems and their different sampling rates. This procedure ensures adaptive and individual noise parameterization for the current user and use case and enables a real-time support.

### 3.2. MARG-Sensor Datafusion

**Figure 2:**
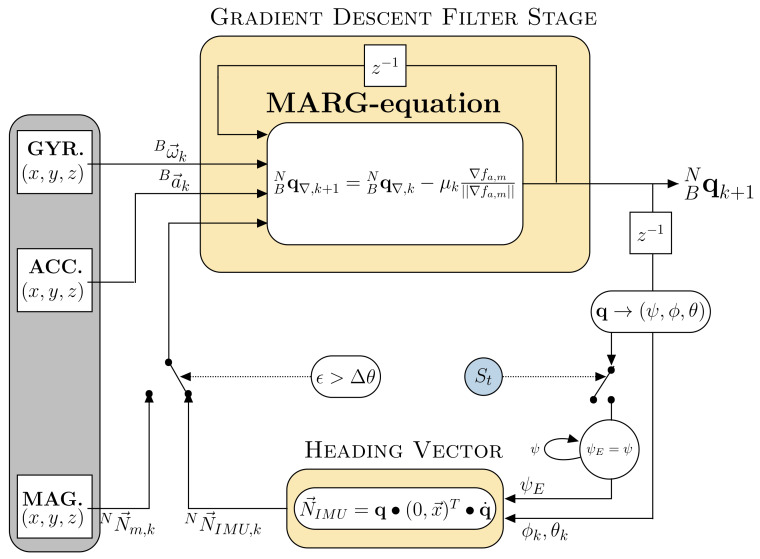
Block diagram of the proposed fusion approach. Upon detection of a zero rotation update through the eye tracker ($S_{t}=1$), the current orientation is used to define an inertial measurement unit (IMU) heading vector (${}^{N}{\vec{N}}_{IMU}$). This IMU heading vector will be updated with accelerometer measurements and fixed heading information as long as $S_{t}=1$ is triggered and magnetic disturbance is present. If magnetic disturbance is present and the trigger is zero ($S_{t}=0$) the IMU heading vector is iterativly updated. Switching between magnetometer based north direction vector (${}^{N}{\vec{N}}_{m}$ ) and IMU heading vector (${}^{N}{\vec{N}}_{IMU}$) is based on the deviation angle ϵ between both vectors.

In general the approach can be used independently of the underlying MARG-sensor data fusion process, since it indicates whether the users head is in dynamic or static motion phases. In this work, we exploit the approach on an adaptation of Madgwick’s gradient descent algorithm (GDA) based filter. Figure 2 depicts the complete filter fusion approach that will be explained in detail in the following subsection.

As proposed by Madgwick et al. a quaternion ${}_{B}^{N}q$ is computed by solving a minimization problem

(3) $$\min_{{}_{B}^{N}q\in R^{4}}\;f{(}_{B}^{N}q,{}^{N}\vec{d},{}^{B}\vec{s}),$$

that rotates a vector ${}^{N}\vec{d}$ into the orientation of a reference vector ${}^{B}\vec{s}$

(4) $$f\left({}_{B}^{N}q,{}^{N}\vec{d},{}^{B}\vec{s}\right)={}_{B}^{N}q•\left(\begin{array}{c} 0\\ {}^{N}\vec{d} \end{array}\right)•{}_{B}^{N}\dot{q}-\left(\begin{array}{c} 0\\ {}^{B}\vec{s} \end{array}\right),$$

where ${}_{B}^{N}$ denotes the orientation of the global navigation frame relative to the body frame and ${}_{B}^{N}q$ is the four component quaternion

(5) $${}_{B}^{N}q={\left(\begin{array}{cccc} q_{1} & q_{2} & q_{3} & q_{4} \end{array}\right)}^{T}.$$

A possible solution to the optimization problem in Equation (3) can be given by gradient descent based solving for the obtained magnetometer and accelerometer vector measurements respectively

(6) $${}_{B}^{N}q_{k+1}={}_{B}^{N}q_{k}-\mu_{t}\frac{\nabla f({}_{B}^{N}q_{k},{}^{N}\vec{d},{}^{B}\vec{s})}{\left||\nabla f({}_{B}^{N}q_{k},{}^{N}\vec{d},{}^{B}\vec{s})|\right|},\;k=0,1,2,\ldots n,$$

where $\mu_{t}$ denotes the stepsize of the gradient function. For a complete mathematical explanation of the filter see Reference [11] or Reference [8]. The GDA filter stage computes a complete quaternion ${}_{B}^{N}q_{k}$ either based on gyroscope, magnetometer and accelerometer (MARG-case) or gyroscope and accelerometer only data (IMU-case). This is to reduce errors in the heading estimate from magnetic disturbances but requires two different sets of equations [8]. This is due to the missing magnetometer measurement vector in the IMU case set of equations and therefore needs an adapted objective function and Jacobian respectively.

In this work, we propose calculating an IMU heading vector that substitutes the magnetometer vector while magnetic disturbance is present to reduce the needed sets of equations as well as to guarantee convergence and a continuous quaternion solution to the minimization problem. We use the north direction vector ${}^{N}{\vec{N}}_{m}$ from the NED formulation through accelerometer and magnetometer measurements while no disturbance is present. The north direction vector is defined as the cross product between the down and east vector,

(7) $${}^{N}{\vec{N}}_{m}{=}^{N}\vec{D}\times^{N}\vec{E},$$

where the down vector is defined as the inverse of the acceleration measurement vector (${}^{B}\vec{a}$)

(8) $${}^{N}\vec{D}={-}^{B}\vec{a},$$

and the east vector is defined as the cross product between the down vector and the magnetometer measurement vector (${}^{B}\vec{m}$)

(9) $${}^{N}\vec{E}{=}^{N}\vec{D}\times^{B}\vec{m}.$$

During rotation the acceleration vector will be subject to motion acceleration and therefore does not accurately reflect the direction of gravity. This effect however is typically reduced by low pass filtering the acceleration vector. Most modern sensors provide onboard low pass filter banks that can be configured by the user to the appropriate needs. Furthermore, the rotational accelerations will be rather small compared to the dominant acceleration originating from gravity. This is due to the small distance (in this case 0.1 m) between the rotational center of the head and the position of the MARG-sensor resulting in minor inaccuracies during dynamic motion. The influence of these incaccuracies during dynamic motion is further reduced by the subsequent data fusion filter. The data fusion filter usually does emphasize gyroscope data integration during fast dynamic motion to reduce inaccuracies from the motion acceleration on the orientation estimation.

We calculate a substitute to the north direction vector, termed IMU heading vector ${}^{N}{\vec{N}}_{IMU}$, based on the orientation estimation from the gyroscope and accelerometer measurements. This is achieved through the following process.

We extract the heading information of the output quaternion ${}_{B}^{N}q_{k}$ by calculating a three component vector (${}^{N}{\vec{N}}_{IMU,k}$) describing heading information in the NED frame. First the heading information (yaw angle, $\psi_{E}$) from the quaternion ${}_{B}^{N}q_{k}$ is converted to Euler angle representation

(10) $$\begin{array}{c} \;a=({q_{k,1}}^{2}+{q_{k,2}}^{2}-{q_{k,3}}^{2}-{q_{k,4}}^{2})\\ \;b=2\cdot (q_{k,2}\cdot q_{k,3}+q_{k,1}\cdot q_{k,4})\\ \psi_{E}=atan2\left(b,a\right). \end{array}$$

When a zero rotation update is triggered, the fusion process samples the current output angle $\psi_{E}$ from the last output quaternion ${}_{B}^{N}q_{k}$ of the GDA filter stage and holds it while the trigger $S_{t}$ is true. The subscript *E* indicates that the angle ψ is not updated if the sample and hold mechanism is activated. If the trigger signal is false, indicating head motion, the fusion process updates the angle $\psi_{E}$ with every new output quaternion ${}_{B}^{N}q_{k}$.

Second we convert the iterative updated roll ($\phi_{k}$) and pitch ($\theta_{k}$) angles derived from the current quaternion ${}_{B}^{N}q_{k}$ by the following process

(11) $$\begin{array}{c} \;a=2\cdot (q_{k,3}\cdot q_{k,4}+q_{k,1}\cdot q_{k,2})\\ \;b=({q_{k,1}}^{2}-{q_{k,2}}^{2}-{q_{k,3}}^{2}+{q_{k,4}}^{2})\\ \;c=2\cdot (q_{k,2}\cdot q_{k,4}+q_{k,1}\cdot q_{k,3})\\ \;\phi_{k}=atan2\left(b,a\right)\\ \;\theta_{k}=asin\left(-c\right). \end{array}$$

From the yaw ($\psi_{E}$) angle and the current roll ($\phi_{k}$) and pitch ($\theta_{k}$) angles we build a new temporary quaternion shown in Equation (12),

(12) $${}_{B}^{N}q_{E,k}=\left[\begin{array}{c} c\left(\frac{\phi_{k}}{2}\right)c\left(\frac{\theta_{k}}{2}\right)c\left(\frac{\psi_{E}}{2}\right)+s\left(\frac{\phi_{k}}{2}\right)s\left(\frac{\theta_{k}}{2}\right)s\left(\frac{\psi_{E}}{2}\right)\\ s\left(\frac{\phi_{k}}{2}\right)c\left(\frac{\theta_{k}}{2}\right)c\left(\frac{\psi_{E}}{2}\right)-c\left(\frac{\phi_{k}}{2}\right)s\left(\frac{\theta_{k}}{2}\right)s\left(\frac{\psi_{E}}{2}\right)\\ c\left(\frac{\phi_{k}}{2}\right)s\left(\frac{\theta_{k}}{2}\right)c\left(\frac{\psi_{E}}{2}\right)+s\left(\frac{\phi_{k}}{2}\right)c\left(\frac{\theta_{k}}{2}\right)s\left(\frac{\psi_{E}}{2}\right)\\ c\left(\frac{\phi_{k}}{2}\right)c\left(\frac{\theta_{k}}{2}\right)s\left(\frac{\psi_{E}}{2}\right)-s\left(\frac{\phi_{k}}{2}\right)s\left(\frac{\theta_{k}}{2}\right)c\left(\frac{\psi_{E}}{2}\right) \end{array}\right],$$

where *c* and *s* are sine and cosine functions respectively.

**Figure 3:**
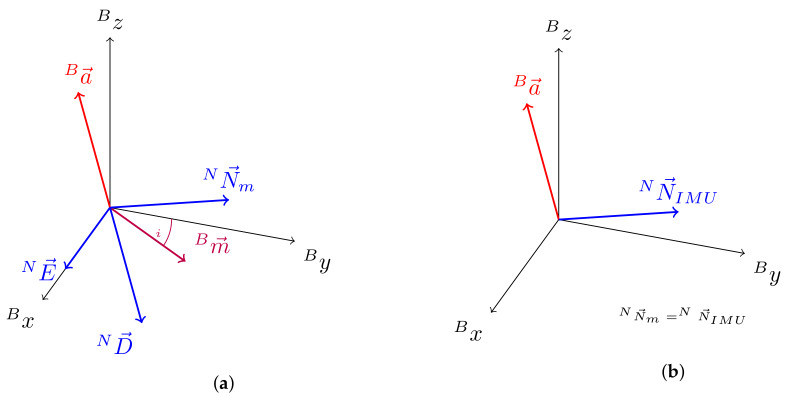
Depiction of the north direction vector substitutes, (**a**) north direction vector, termed ${}^{N}{\vec{N}}_{m}$ from accelerometer (${}^{B}\vec{a}$) and magnetometer (${}^{B}\vec{m}$) measurements in the case of undisturbed magnetic field measurement, and (**b**) IMU heading vector, termed ${}^{N}{\vec{N}}_{IMU}$ calculated based on quaternion vector multiplication from the gradient descent algorithm (GDA) filter without magnetometer data in the case of disturbed magnetic field measurements.

This quaternion is now applied to a x-axis unit vector because the north direction vector defines the sensors body x-axis, resulting in

(13) $$\begin{array}{c} \vec{x}={\left(\begin{array}{ccc} 1 & 0 & 0 \end{array}\right)}^{T}\\ {}^{N}{\vec{N}}_{IMU,k}{=}_{B}^{N}q_{E,k}•\left(\begin{array}{c} 0\\ \vec{x} \end{array}\right){•}_{B}^{N}{\dot{q}}_{E,k} \end{array},$$

where • indicates quaternion multiplication and $\dot{q}$ represents the conjugate quaternion to q. The vector ${}^{N}{\vec{N}}_{IMU,k}$ now represents the direction as a substitute to the magnetometer based north direction vector in the NED frame, as can be seen in Figure 3.

Since the vectors ${}^{N}{\vec{N}}_{m}$ and ${}^{N}{\vec{N}}_{IMU}$ lie in the same plane it is possible to calculate a deviation angle (ϵ) that can be used to determine magnetic disturbance due to sudden changes in the direction of the north direction vector in contrast to the IMU heading vector. The deviation angle is calculated as follows

(14) $$\epsilon =cos^{-1}\left({}^{N}{\vec{N}}_{m}{•}^{N}{\vec{N}}_{IMU}\right),$$

where • represents the dot product respectively.

If magnetic disturbance is present, the deviation angle ϵ will increase. If it exceeds a threshold Δθ, the filter switches towards the virtual sensor vector based quaternion calculation and vice versa if it vanishes. This procedure enables the calculation of a complete and continuous quaternion solution that involves current sensor measurements from the accelerometer and the extracted heading information from the previous quaternion. It is possible to use the same set of equations without any adaptation and switch from magnetometer based north direction vector to the IMU heading vector without divergence of the quaternion. While the zero rotation trigger is enabled, the fusion process holds the recent calculated yaw angle $\psi_{E}$. This ensures that the GDA based calculation of the new quaternion ${}_{B}^{N}q_{k}$ is less affected by possible drift in the heading direction due to uncorrected gyroscope bias but will however be corrected in the remaining axes through accelerometer updates and preserves a continuous solution and convergence. While no trigger is emitted, the fusion approach simply updates the measurement quaternion with every iteration based on either magnetic north direction vector when no disturbance is present or the IMU heading vector from the current orientation. It is known that Euler angle representation is subject to gimbal lock if two rotation axis align. This effect can be dealt with in two different ways. Either by designing the experiment in a way that does not include head rotations around the pitch exceeding $\pm 90^{\circ }$, which causes gimbal lockin the chosen rotation order (Z-Y-X), or by formulating a quaternion based heading orientation estimation method. The quaternion based solution can be found in the following paragraph.

The heading information (yaw angle, $\psi_{E}$) from the quaternion ${}_{B}^{N}q_{k}$ is converted to a quaternion representing only the yaw rotation (${}_{B}^{N}q_{\psi ,E}$) by deriving it from the corresponding Euler angle representation [23]. A unit quaternion representing heading information (${}_{B}^{N}q_{\psi }$) is expressed as a rotation ψ around the z-axis

(15) $$\begin{array}{c} q=\left(\begin{array}{cccc} cos(\psi /2) & 0 & 0 & {sin\left(\psi /2\right))}^{T} \end{array}\right),\\ {}_{B}^{N}q_{\psi }=\frac{q}{∥q∥}. \end{array}$$

We can express the heading quaternion ${}_{B}^{N}q_{\psi ,E}$ without trigonometric functions by substituting the corresponding Euler angle Equation (10) with (15) and normalize it, resulting in

(16) $$\begin{array}{c} q={\left(\begin{array}{cccc} \left(\left({q_{k,1}}^{2}+{q_{k,2}}^{2}-{q_{k,3}}^{2}-{q_{k,4}}^{2}\right)\right) & 0 & 0 & \left(2\cdot \left(q_{k,2}\cdot q_{k,3}+q_{k,1}\cdot q_{k,4}\right)\right) \end{array}\right)}^{T},\\ {}_{B}^{N}q_{\psi }=\frac{q}{∥q∥}. \end{array}$$

To get the half rotation angle from Equation (10) we add a unit quaternion and normalize the result

(17) $$\begin{array}{c} q{=}_{B}^{N}q_{\psi }+{\left(\begin{array}{cccc} 1 & 0 & 0 & 0 \end{array}\right)}^{T},\\ {}_{B}^{N}q_{\psi ,E}=\frac{q}{∥q∥}. \end{array}$$

Likewise, to the Euler angle solution the zero rotation update trigger samples the current output quaternion ${}_{B}^{N}q_{\psi ,E}$ from the last output quaternion ${}_{B}^{N}q_{k}$ of the GDA filter stage and holds it while it is activated. If the trigger signal is deactivated, the fusion process updates the heading quaternion ${}_{B}^{N}q_{\psi ,E}$ with every new output quaternion ${}_{B}^{N}q_{k}$.

We calculate a quaternion (${}_{B}^{N}q_{\phi ,\theta ,k}$) representing the iterative updated roll ($\phi_{k}$) and pitch ($\theta_{k}$) angles based on the current quaternion ${}_{B}^{N}q_{k}$. This is achieved by removing the yaw component of the current quaternion ${}_{B}^{N}q_{k}$ through conjugate quaternion multiplication. We calculate a yaw quaternion ${}_{B}^{N}q_{\psi ,k}$ based on the Equation (16) and apply the conjugate to the current quaternion ${}_{B}^{N}q_{k}$

(18) $${}_{B}^{N}q_{\phi ,\theta ,k}{=}_{B}^{N}{\dot{q}}_{\psi ,k}{•}_{B}^{N}q_{k},$$

where • indicates quaternion multiplication and $\dot{q}$ represents the conjugate quaternion to q.

The final quaternion ${}_{B}^{N}q_{E,k}$ can be computed by combining the heading quaternion ${}_{B}^{N}q_{\psi ,E}$ and the iterative updated quaternion representing only roll and pitch ${}_{B}^{N}q_{\phi ,\theta ,k}$ through quaternion multiplication,

(19) $${}_{B}^{N}q_{E,k}{=}_{B}^{N}q_{\psi ,E}{•}_{B}^{N}q_{\phi ,\theta ,k}.$$

The quaternion ${}_{B}^{N}q_{E,k}$ now represents a complete orientation expressed as quaternion and combines heading information from the sample and hold mechanism with current updates regarding roll and pitch information from the filter’s output quaternion. This solution does not suffer from gimbal lock and can be used as the input quaternion to Equation (13). Both methods, Euler angle conversion or the complete quaternion based heading calculation, are valid and can be chosen based on the desired application and design of experiment.

## 4. Interface Setup

**Figure 4:**
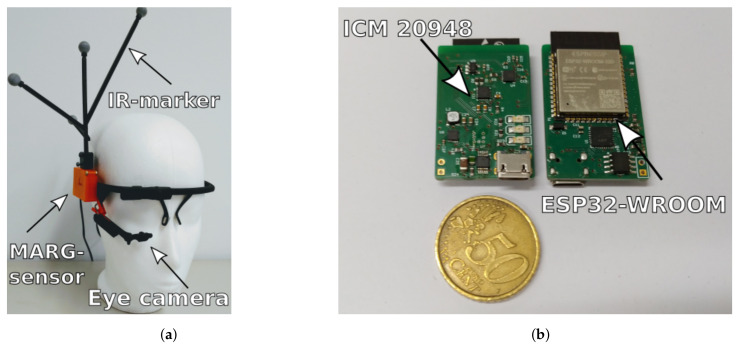
Proposed head interface and custom designed MARG-sensor board. The head interface (**a**) consists of a pupil core headset, a passive IR-marker tree that is in line to the custom MARG-sensor board (**b**) which is attached to the eye tracker frame.

We use a custom designed MARG-sensor board running the GDA based sensor data fusion on an Espressif 32 bit dual-core microcontroller unit (MCU). The sensor board features a low power 9-axis ICM 20948 InvenSense MARG-sensor, see Figure 4. The MCU is running the FreeRTOS realtime operating system on both cores at 1 kHz scheduler tick rate [24]. The standalone implementation is designed to simultaneous calculate orientation data from two copies of the data fusion process at 250 Hz, while their only difference is the active eye tracking trigger update. The two data fusion filters run in real time on the MCU and publish the two sets of fused orientation data and the calibrated 9-axis sensor data are at 100 Hz via micro-USB over UART. The sensor is attached via a custom casing to a low cost monocular eye tracker from Pupil Labs [19]. The tracker frame is secured via an eyewear strap on the users head. The eye tracker features 120 Hz frame rate of the eye tracking process at a resolution of 400×400 pixels. It is connected to a host computer running the Pupil Labs open source capture tool to acquire and preprocess the data as well as taking care of online fixation detection. The data are accessible in real-time through ZeroMQ. Two custom c++ ROS (Robot Operating System) nodes handle the synchronization and inter device communication. Synchronization between the MARG and eye tracking data is achieved through comparison of their corresponding timestamp upon arriving at the host system with a maximum lag of 3 ms between the timestamps. While fixation is true, the trigger signal is broadcasted to the MARG-sensor system indicating zero rotation. Furthermore, the trigger starts the gyroscope raw data capture process on the host computer. When the visual fixation is released the trigger is set to false which stops the gyroscope capture process as well as the zero orientation update cycle. The median gyroscope noise for stationary motion phases is sent to the MARG-sensor, when sufficient amount of data has been captured. To reduce latency impact on the orientation calculation, a movement threshold based on this median gyroscope noise is implemented on the MARG-sensor to ensure that the trigger will be set to false without latency drops or desynchronization.

## 5. Experimental Setup

The accuracy of the proposed interface is benchmarked against a Qualisys motion capture system (Qualisys Miqus Camera M3, Qualisys AB, Kvarnbergsgatan 2, Göteborg, Sweden). The interface is worn by a user alongside a leightweight medium density fiberboard based rigid body passive IR-marker tree connected to the MARG-sensor casing, see Figure 4. The capture process of the Qualisys motion capture system broadcasts data at 120 Hz over a real-time client, allowing for timestamp based synchronization via the before mentioned ROS-nodes.The threshold Δθ is chosen based on the 3σ standard deviation of the north direction vector under static conditions for a short period of time (10 s). The first calculated north direction vector of this series is the initial vector. This initial vector is used to calculate the standard deviation of this series of deviation angles ϵ based on Equation (14). For the ICM20948 on the custom sensor board the threshold Δθ results in $3^{\circ }$. After a warm up phase, the magnetometer data is turned off to simulate a magnetically disturbed environment and examine the eye tracking supported zero orientation change trigger update mechanism as a proof of concept. The proof of concept of the proposed orientation estimation update mechanism can be provided either by using real magnetometer data or by turning off the magnetometer data measurement completely. A difference is not evident. This is due to the switching from north heading vector to IMU heading vector when a magnetic disturbance is present. In such a case magnetometer data is not used in the orientation estimation algorithm and is therefore not dependent on real magnetometer data input. Thus, we simulate disturbance by switching magnetometer data off to investigate the performance of the proposed data fusion during periods of non usable magnetometer input. To compare rotations, the coordinate system of the Qualisys data is transformed into the body coordinate frame of the MARG-sensor by calculating an alignment matrix from six stationary positions through least square method described in Reference [25]. The user is instructed to freely move his head and eyes with some static or no-rotation phases spanning between 2–5 s in duration. The total duration for one trial was limited to 15 min. A total of six trials was gathered for one individual user as a proof of concept for the proposed method. Visual fixation detection parameters were chosen based on experimental pretests that minimize latency drops when changing from stationary to dynamic head motion and were set to the following: $th_{d}=0.21{}^{\circ}$, and $th_{t}=220$ ms.

Two pretests were conducted to demonstrate the interchangeability of the north direction vector substitute calculations described in Section 3.2 and the filter’s capability to detect interference based on the deviation angle ϵ. The three axis magnetometer was calibrated based on the process described in Reference [26]. The MARG-sensor is rotated arbitrarily in all dimensions. The tri-axis magnetometer data is sampled during this period. After recording the magnetometer data is calibrated through least square fitting of the ellipsoid data into a unit sphere and scaled to the surrounding field strength afterwards. During a 10 min warm up phase, the filter uses magnetometer data to converge towards the direction of magnetic north and gravity respectively. To demonstrate the interchangibility of the vectors we switch off the filter to use the IMU heading vector instead of the magnetometer based north direction vector and move the sensor arbitrarily for a short period of time (50 s). Both vector measurements are recorded throughout the trial. The second pretest covers the validation of magnetic interference detection and the switching from north to IMU heading vector. The sensor is set up according to the previous mentioned calibration and warm up routines. We record two sets of orientation: (a) The proposed filter with magnetic interference detection and switching and (b) the same filter without the switching mechanism. After 44 s an iron bar is brought close to the sensor (15 cm) to introduce magnetic interference.

## 6. Results and Discussion

In this work it is proposed to support MARG-sensor based heading estimates by zero rotation updates measured and indicated by visual fixation detection. An interchangeable north direction vector substitute is used for a gradient descent based orientation estimation. Section 6.1 gives an overview of the pretest to show the interchangeability of the heading vector substitutes calculation described in Section 3.2 whereas Section 6.2 the filters capability to detect magnetic disturbance and switch towards IMU heading vector. Section 6.3 presents the experimental results from the full fusion approach using visual fixations for zero rotation update.

### 6.1. Interchangeable North Direction Vector Substitutes

**Figure 5:**
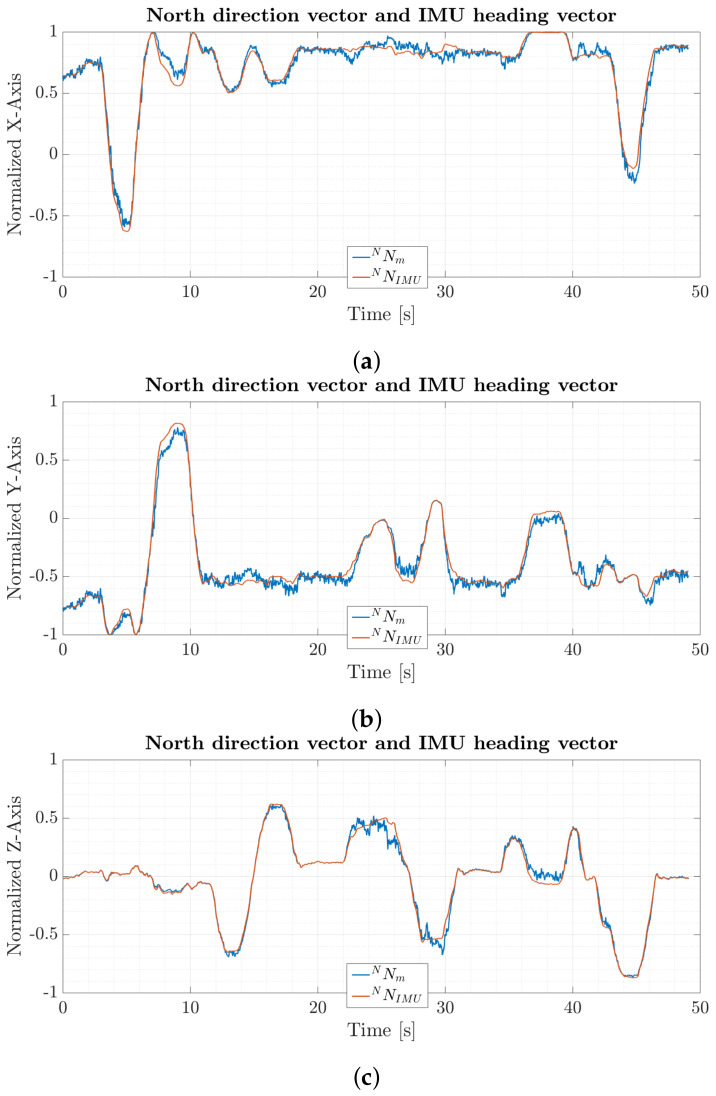
Comparison between (**a**) magnetometer based normalized x-axis north direction vector and normalized x-axis IMU heading vector, (**b**) magnetometer based normalized y-axis north direction vector and normalized y-axis IMU heading vector and (**c**) magnetometer based normalized z-axis north direction vector and normalized z-axis IMU heading vector.

Figure 5 depicts normalized individual x-, y- and z-axis results for north direction vector ${}^{N}{\vec{N}}_{m}$ from calibrated magnetometer data through Equations (7)–(9) and the IMU heading vector ${}^{N}{\vec{N}}_{IMU}$ based on the process given by Equations (10)–(13). Both vectors show similar results during the whole trial with maximum deviations of ±0.1 normalized units. The north direction vector from magnetometer data has a larger spread of measurement values compared to the IMU heading vector. This originates from the different noise characteristics and computations of the vector components. The north direction vector is directly calculated from raw accelerometer and magnetometer data and will directly reflect raw sensor noise, whereas the IMU heading vector is based on smoothed quaternion fusion from gyroscope and accelerometer readings from the GDA filter. The noise spreading level, however, does stay at a reasonable level during the trial. This pretest shows the interchangeability of the different north direction vector substitutes which guarantees a continuous quaternion solution and convergence of the filter.

The IMU heading vector will drift apart from the north direction vector with respect to time. This is due to uncorrected gyroscope biasresulting in drift in the heading estimate of the quaternion used for determining the IMU heading vector. For short periods of time and under the same initial conditions however both vectors are almost identical. The length of the time period in which both vectors are mostly identical depends on the individual noise characteristics of the used gyroscope and computational errors from the discrete implementation. High grade navigation gyroscopes will experience less drift compared to consumer based gyroscopes used in this work. The maximum time before gyroscope errors accumulate more than $1^{\circ }$ drift in the heading estimate is 50 s for the custom MARG-sensor board used in this work.

### 6.2. Magnetic Disturbance Detection

**Figure 6:**
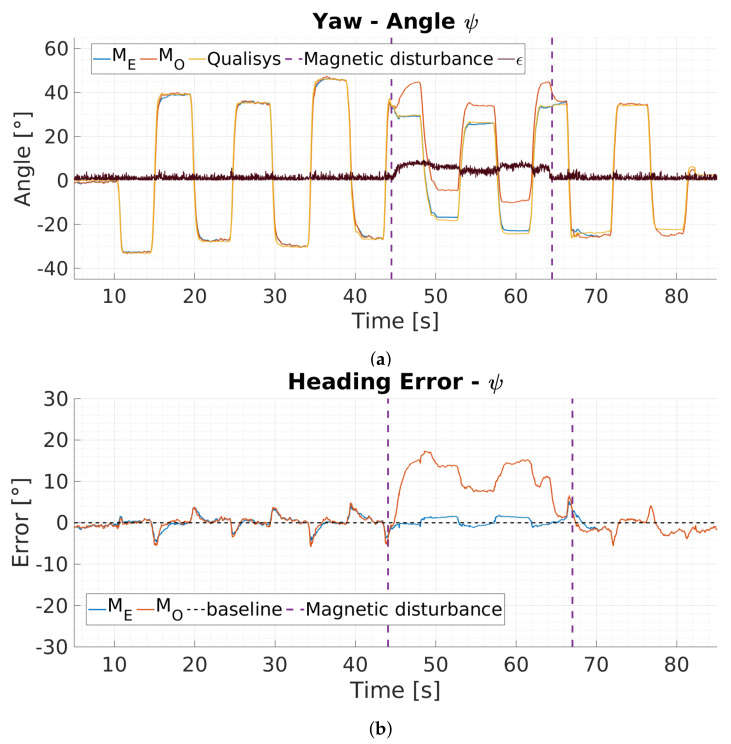
Influence of magnetic disturbance on yaw angle estimation: (**a**) yaw angle comparisons between ground truth (Qualisys, yellow), the proposed filter with magnetic disturbance detection and heading vector substitute switching (*M_E_*, blue), a filter version without heading vector switching (*M_O_*, orange) as well as the magnetic deviation angle e (black). (**b**) depicts the corresponding heading error referenced to the Qualisys system. Magnetic interference is introduced for a short period of time (43 s to 66 s) by bringing and iron bar close to the MARG-sensor. The proposed filter detects the interference and switches towards IMU heading vector usage resulting in less error.

Figure 6 depicts yaw angle results as well as the corresponding yaw angle errors for the magnetic disturbance detection and switching from north direction to IMU heading vector based on Equation (14). The Figure 6a presents three different yaw angle estimations over time: ground truth yaw data (Qualisys, yellow), yaw estimations from the proposed filter with deviation detection and heading vector substitutes ($M_{E}$, blue) as well as yaw estimations of a version of the filter without heading vector switching ($M_{O}$, orange). The figure also presents values for the magnetic deviation angle ϵ (black) over time. Figure 6b presents the corresponding yaw angle errors referenced to the ground truth yaw data. Magnetic disturbance is introduced for a short period of time starting at 44 s and ending at 66 s by bringing an iron bar close to the sensor (15 cm).

The proposed filter ($M_{E}$) detects the disturbance when it is introduced because the deviation angle ϵ exceeds the threshold Δθ, see Figure 6a. The filter switches towards IMU heading vector substitute and is not affected by the disturbance, resulting in a maximum error of $2^{\circ }$ during this phase. In contrast, the filter without switching mechanism experiences large yaw angle erros and results in up to $17^{\circ }$ total error (see Figure 6b). This pretest demonstrates the filters capability of magnetic disturbance detection based on the deviation angle calculation between north direction and IMU heading vector. After the filter detects a disturbance it uses the IMU heading vector for orientation estimation. In this mode the filter furthermore enables visual zero rotation updates mechanism to reduce heading error accumulation over time.

### 6.3. MARG-Sensor Data Fusion Approach Using Visual Fixations

**Figure 7:**
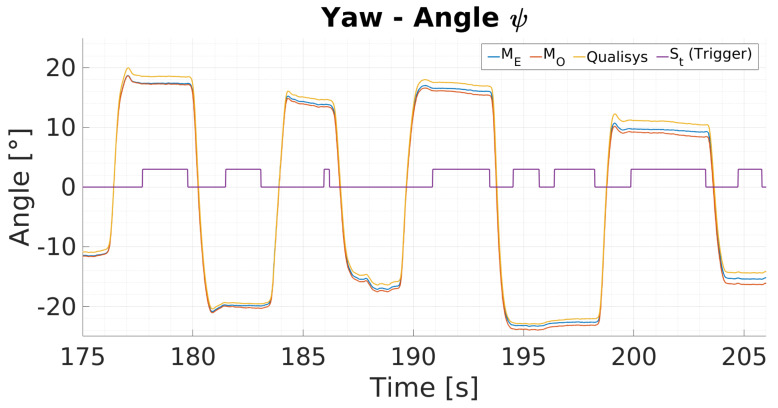
Typical yaw angle measurements for a motion sequence from the Qualisys system (yellow), the proposed ($M_{E}$, blue) and standard version ($M_{O}$, orange) of the data fusion. During stationary motion, the trigger signal $S_{t}$ (purple) is set to high and indicates zero orientation change.

Figure 7 shows typical data for yaw angle measurements from a 30 s sequence of one 900 s trial. The yaw angles are presented in degrees over time in seconds. The figure depicts yaw angle estimation data from the ground truth motion capture system (Qualisys system, yellow), the proposed ($M_{E}$, blue) and standard version ($M_{O}$, orange) of the data fusion process. The visual fixation trigger state $S_{t}$ is presented in purple. During visual fixation phases ($S_{t}$, purple) the proposed eye tracking supported version $M_{E}$ does drift less compared to the standard implementation $M_{O}$. In dynamic motion phases both filter versions do accumulate the same amount of drift.

**Figure 8:**
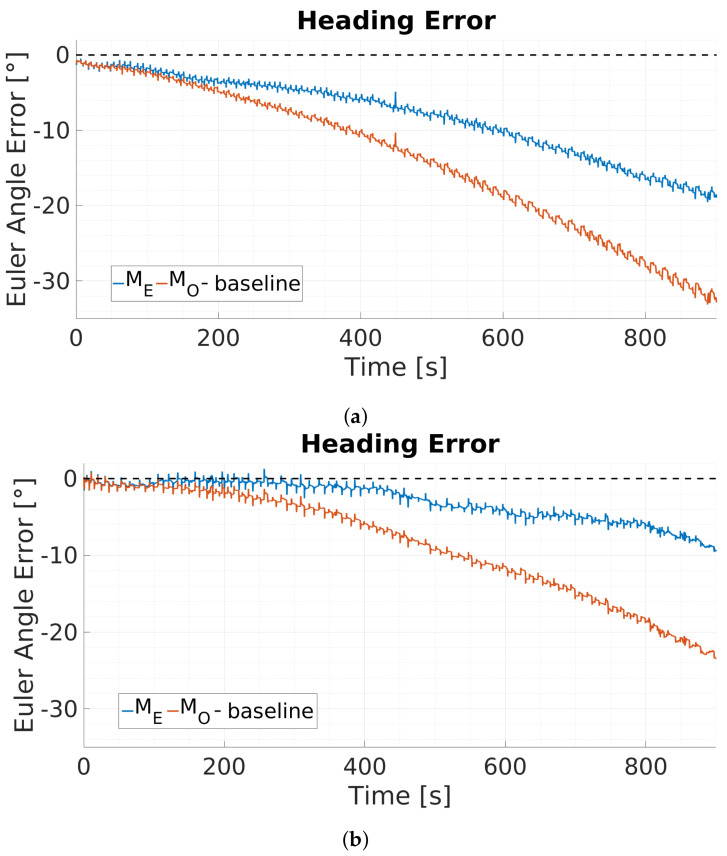
Typical results for absolute heading error referenced to the Qualisys system from the GDA based approach with (*M_E_*, blue) and without eye tracking (*M_O_*, orange) support for two different trials. The eye tracking supported filter results in (**a**) a total of 18.84° error whereas the standard version results in 32.76° and (**b**) in a total of 8.82° error for the eye tracking supported version whereas the standard version results in 22.55°.

The performance of the proposed approach for the heading estimation is presented as total Euler angle error (degrees see Figure 8) as well as mean error reduction rate ($e_{\psi }$, unitless see Table 1). The total Euler angle errors are calculated as the difference between the ground truth of absolute orientation from the Qualisys system and the orientation estimation of the proposed ($M_{E}$) and standard version ($M_{O}$) of the data fusion process (see Figure 7). The dashed black line indicates the reference for zero heading error. Figure 8 presents two sets of Euler angle errors in degrees over time for the complete 900 s duration of two different trials. At the start of the trial the eye tracking supported version of the filter ($M_{E}$, blue) as well as the standard GDA filter ($M_{O}$, orange) perform identical. During dynamic phases both filters accumulate the same amount of error due to uncorrected gyroscope bias. However, when stationary phases are indicated and the trigger signal $S_{t}$ is enabled, the eye tracker supported GDA filter version accumulates less gyroscope drift in contrast to the standard implementation, see Figure 8. This effect covers the entire duration of the trials. The orientation estimation errors rise significantly over time for both solution. The GDA based approach with eye tracking based zero orientation change update results in nearly 50% less absolute orientation, see Figure 8.

The mean error reduction rate $e_{\psi }$ and its standard deviation is calculated based on the absolute error quotient between the proposed ($M_{E}$) and standard version ($M_{O}$) of the fusion process at 900 s. Table 1 presents total Euler angle errors from six different trials for the proposed ($M_{E}$) and standard version ($M_{O}$) of the fusion after 900 s and the calculated error reduction rate. On average, the eye tracking supported GDA filter approach accumulates near 50% less orientation error (0.46±0.17) compared to the GDA filter without eye tracking data support, see Table 1.

## 7. Conclusions

Utilizing eye tracking data to support sensor data fusion of MARG-sensor shows improvements of the heading accuracy in magnetically disturbed environments or for IMU sensors that do not feature a heading reference in the order of 50%. Because of the physiological coupling between eye and head rotations, eye tracking can deliver an indicator signal for near zero head orientation change. Furthermore, this trigger signal allows for individual and adaptive noise parameterization through gyroscope capturing and could be used in the context of adaptive noise estimation with respect to head motion while a sufficient amount of data is captured. The proposed method can be used with any mobile eye tracking devices that either feature a build-in IMU or MARG-sensor or are expanded by a custom or third-party sensor. The presented approach does not need a world camera and is therefore mostly independent of surrounding environmental lightning conditions. In addition, the proposed use of interchangeable north direction vector substitutes enables switching between full MARG and IMU-mode, without the need for an additional set of equations in a given filter. This guarantees a continuous quaternion computation and convergence of the filter.

### Limitations

The magnitude of error compensation does scale with respect to total fixation duration and amount of stationary motion phases. However, the solution does not reduce the effect during dynamic motion phases, since it does not directly estimate and correct the dc-bias term of the raw gyroscope signal. This is due to various other noise effects that are present in the raw gyroscope signal. Main noise terms among other that influence the in-run dc-bias estimation are ac-noise, oscillating head motion, output rate limitations and possible desynchronization between timestamps of both systems.

When estimating in-run dc-bias the presence of these noise terms can lead to a wrong estimation. Since the dc-bias is subtracted from the raw gyroscope signal at every time step, it effects the complete measurement from that point forward and might result in a worse heading estimate. However, if a sufficient amount of sensor data has been gathered, a low-pass filtered dc-bias estimation might be used to reduce the drift at a smaller scale since the data are only captured during near stationary motion phases and therefore restrain heavy amplitude changes.

The proposed solution can be affected by very slow motion acceleration triggering the visual fixation detection plugin and falsely labeling a static phases. This effect however only appears if the resulting angular rate of the head motion is smaller than the angular rate constraint derived from the dispersion and time threshold of the fixation detection plugin and stays below the median angular rate threshold that is sampled throughout the trial. In this work the angular rate constraint from the fixation detection plugin that might lead to a wrong classification during fixation and simulatanious head motion is $0.95\;\frac{\circ }{s}$ for a 220 ms measurement window. This angular rate results in the maximum dispersion of 0.21°. This would result in a fixation detection which would in turn trigger the zero rotation update mechanism for one cycle. After this the dispersion threshold is exceeded, setting the trigger to false which in turn resets the online fixation detection sliding window.

## 8. Future Work

Future research will focus on adaptive gyroscope noise parameter estimation based on the proposed visual fixation trigger for head motion detection. The gyroscope noise parameter estimation can be used to reduce the heading errors even further and without the visual fixation trigger being active. While a sufficient amount of samples is gathered during visual fixations an adapted noise parameter can be estimated and used to identify no motion phases just as the visual fixation trigger.

A second instance of the filter running in parallel could be used to compute orientation that includes the estimated gyroscope noise and compare it to the first instance of the filter in real-time. Based on the deviation between both solutions, the estimated bias could be used or discarded from that point on which in turn will lead to improved heading accuracy. Advanced parameter specification of the proposed fusion method will be explored by a broader set of experiments, including experiments in real use cases, multiple age varying paticipants as well as the influence of gyroscope noise parameter estimation on the proposed method.

## Figures and Tables

**Table 1:** Absolute error values and error reduction rate ($e_{\psi }$) for the GDA based data fusion with ($M_{E}$) and without eye tracking ($M_{O}$) support after 900 s.

	$M_{E}\;{[}^{\circ }]$	$M_{O}\;{[}^{\circ }]$	$e_{\psi }=1-\frac{M_{E}}{M_{O}}$
1.	−18.84	−32.76	0.42
2.	−8.82	−22.55	0.59
3.	−13.89	−23.17	0.40
4.	−11.33	−19.09	0.40
5.	−12.63	−26.06	0.51
6.	−7.16	−13.52	0.45
average			0.46±0.17

## Abbreviations

The following abbreviations are used in this manuscript:

MARG	MagneticAngularate Gravity sensor
IMU	Inertial Measurement Unit
GDA	Gradient Descent Algorithm
MEMS	Micro-Electro-Mechanical Systems
API	Application Programming Interface
NED	North East Down
MCU	Microcontroller Unit
dc-bias	direct current bias

## References

[1] Rudigkeit N., Gebhard M. (2019). AMiCUS—A Head Motion-Based Interface for Control of an Assistive Robot. Sensors.

[2] Jackowski A., Gebhard M., Thietje R. (2018). Head Motion and Head Gesture-Based Robot Control: A Usability Study. IEEE Trans. Neural Syst. Rehabil. Eng..

[3] Alsharif S., Kuzmicheva O., Gräser A. (2016). Gaze Gesture-Based Human Robot Interface. Technische Unterstützungssysteme die die Menschen Wirklich Wollen.

[4] Wendel J. (2011). Integrierte Navigationssysteme: Sensordatenfusion, GPS und Inertiale Navigation.

[5] Shiau J.K., Huang C.X., Chang M.Y. (2012). Noise characteristics of MEMS gyro’s null drift and temperature compensation. J. Appl. Sci. Eng..

[6] Valenti R.G., Dryanovski I., Xiao J. (2016). A linear Kalman filter for MARG orientation estimation using the algebraic quaternion algorithm. IEEE Trans. Instrum. Meas..

[7] Gebre-Egziabher D., Elkaim G., Powell J.D., Parkinson B. A non-linear, two-step estimation algorithm for calibrating solid-state strapdown magnetometers. Proceedings of the 8th St. Petersburg International Conference on Integrated Navigation Systems (IEEE/AIAA).

[8] Wöhle L., Gebhard M. A robust quaternion based Kalman filter using a gradient descent algorithm for orientation measurement. Proceedings of the 2018 IEEE International Instrumentation and Measurement Technology Conference (I2MTC).

[9] Sabatini A.M. (2006). Quaternion-based extended Kalman filter for determining orientation by inertial and magnetic sensing. IEEE Trans. Biomed. Eng..

[10] Roetenberg D., Luinge H., Slycke P. (2009). Xsens MVN: Full 6DOF Human Motion Tracking Using Miniature Inertial Sensors.

[11] Madgwick S.O., Harrison A.J., Vaidyanathan R. Estimation of IMU and MARG orientation using a gradient descent algorithm. Proceedings of the IEEE International Conference on Rehabilitation Robotics (ICORR).

[12] Robert-Lachaine X., Mecheri H., Larue C., Plamondon A. (2017). Effect of local magnetic field disturbances on inertial measurement units accuracy. Appl. Ergon..

[13] Wu Z., Sun Z., Zhang W., Chen Q. (2015). Attitude and gyro bias estimation by the rotation of an inertial measurement unit. Meas. Sci. Technol..

[14] Wu Y., Zou D., Liu P., Yu W. (2018). Dynamic Magnetometer Calibration and Alignment to Inertial Sensors by Kalman Filtering. IEEE Trans. Control Syst. Technol..

[15] Aqel M.O., Marhaban M.H., Saripan M.I., Ismail N.B. (2016). Review of visual odometry: Types, approaches, challenges, and applications. SpringerPlus.

[16] Valenti R., Sebe N., Gevers T. (2012). What are you looking at?. Int. J. Comput. Vis..

[17] Kassner M., Patera W., Bulling A. (2014). Pupil: An Open Source Platform for Pervasive Eye Tracking and Mobile Gaze-based Interaction. Adjunct Proceedings of the 2014 ACM International Joint Conference on Pervasive and Ubiquitous Computing.

[18] Wöhle L., Miller S., Gerken J., Gebhard M. A Robust Interface for Head Motion based Control of a Robot Arm using MARG and Visual Sensors. Proceedings of the 2018 IEEE International Symposium on Medical Measurements and Applications (MeMeA).

[19] Pupil Labs GmbH Pupil Core. Open Source Eye Tracking Platform Home Page. https://pupil-labs.com/products/core/.

[20] Larsson L., Schwaller A., Holmqvist K., Nyström M., Stridh M. Compensation of head movements in mobile eye-tracking data using an inertial measurement unit. Proceedings of the 2014 ACM International Joint Conference on Pervasive and Ubiquitous Computing: Adjunct Publication.

[21] Salvucci D.D., Goldberg J.H. Identifying fixations and saccades in eye-tracking protocols. Proceedings of the 2000 Symposium on Eye Tracking Research & Applications.

[22] Duchowski A.T., Jörg S. (2016). Eye animation. Handbook of Human Motion.

[23] Diebel J. (2006). Representing attitude: Euler angles, unit quaternions, and rotation vectors. Matrix.

[24] Real Time Engineers Ltd. FreeRTOS. Real-Time Operating System for Microcontrollers. https://www.freertos.org/Documentation/RTOS_book.html.

[25] STMicroelectronics Parameters and Calibration of a Low-g 3-Axis Accelerometer, Application Note 4508. https://www.st.com/resource/en/application_note/dm00119044-parameters-and-calibration-of-a-lowg-3axis-accelerometer-stmicroelectronics.pdf.

[26] Gebre-Egziabher D., Elkaim G.H., David Powell J., Parkinson B.W. (2006). Calibration of strapdown magnetometers in magnetic field domain. J. Aerosp. Eng..

